# A Complex Presentation of Acute Pancreatitis With HELLP Syndrome: A Case Report and Literature Review

**DOI:** 10.7759/cureus.88868

**Published:** 2025-07-27

**Authors:** Ans Alamami, Rabee Tawel, Ezzeddin Ibrahim, Abdurrahmaan Elbuzidi, Habib Rehman

**Affiliations:** 1 Critical Care Medicine, Hamad Medical Corporation, Doha, QAT

**Keywords:** aki, hellp syndrome, pancreatitis, preeclampsia, pregnancy

## Abstract

HELLP syndrome, defined by hemolysis, elevated liver enzymes, and low platelets, is a life-threatening complication of preeclampsia that rarely coincides with acute pancreatitis during pregnancy. This combination poses profound risks for both mother and fetus.

We describe a 29-year-old woman at 27 weeks of gestation who presented with severe abdominal pain, vomiting, and a history of hypertension. Laboratory evaluation revealed anemia, thrombocytopenia, elevated liver enzymes, renal dysfunction, and high amylase and lipase, confirming the diagnosis of HELLP syndrome associated with acute pancreatitis. Intensive care management included hemodynamic support, electrolyte correction, blood product transfusion, antihypertensive therapy, and an emergency cesarean section. Both maternal and neonatal outcomes were favorable. This case underscores the importance of early multidisciplinary intervention and comprehensive diagnostic evaluation when HELLP syndrome presents with atypical features such as pancreatitis. Prompt recognition and coordinated management are crucial to optimizing maternal and fetal outcomes in these rare and serious presentations.

## Introduction

HELLP syndrome, characterized by hemolysis, elevated liver enzyme levels, and low platelet counts, represents a severe and multifaceted spectrum of preeclampsia. Although its hepatic and hematological complications are well-recognized, they may be complicated by acute pancreatitis, which is a rare but formidable challenge, especially during pregnancy. The concurrence of these conditions dramatically increases both maternal and fetal risks. When encountered in patients with underlying comorbidities, such as hypertension and substance use disorders, management becomes even more demanding. In this report, we discuss the complex presentation of HELLP syndrome and acute pancreatitis in the third trimester, emphasizing clinical nuances and a literature-informed perspective on its pathophysiology and management [1]. HELLP syndrome represents a severe and potentially fatal manifestation of preeclampsia, occurring in approximately 0.5-0.9% of all pregnancies and up to 10-20% of those complicated by severe preeclampsia [1,2]. The syndrome is characterized by endothelial dysfunction and microangiopathic hemolysis, resulting in multiorgan failure. While hepatic, renal, and hematological complications are well-recognized, pancreatic involvement in the form of acute pancreatitis is exceedingly rare, with only isolated reports in the literature [3,4]. Acute pancreatitis in pregnancy is estimated to occur in 1 in 1,000 to 1 in 12,000 pregnancies, mostly late in pregnancy [5]. The predominant etiologies are gallstones and hypertriglyceridemia, while cases secondary to preeclampsia or HELLP syndrome remain uncommon [6]. The occurrence of HELLP syndrome with acute pancreatitis represents a distinctive diagnostic and therapeutic challenge, particularly in women with underlying comorbidities such as hypertension. This case report aims to highlight the clinical complexities of concurrent HELLP syndrome and acute pancreatitis in pregnancy. We provide a detailed description of the presentation, diagnostic process, and management, followed by a review of the current literature to highlight the multidisciplinary approach in similar scenarios.

## Case presentation

A 29-year-old woman, gravida 5 para 4, at 27 weeks of gestation, presented to the emergency department with severe, persistent abdominal pain radiating to the back, accompanied by repeated vomiting, palpitations, and chest discomfort. She denied fever, respiratory, or urinary symptoms. Her medical history was notable for controlled hypertension and asthma.

**Figure 1:**
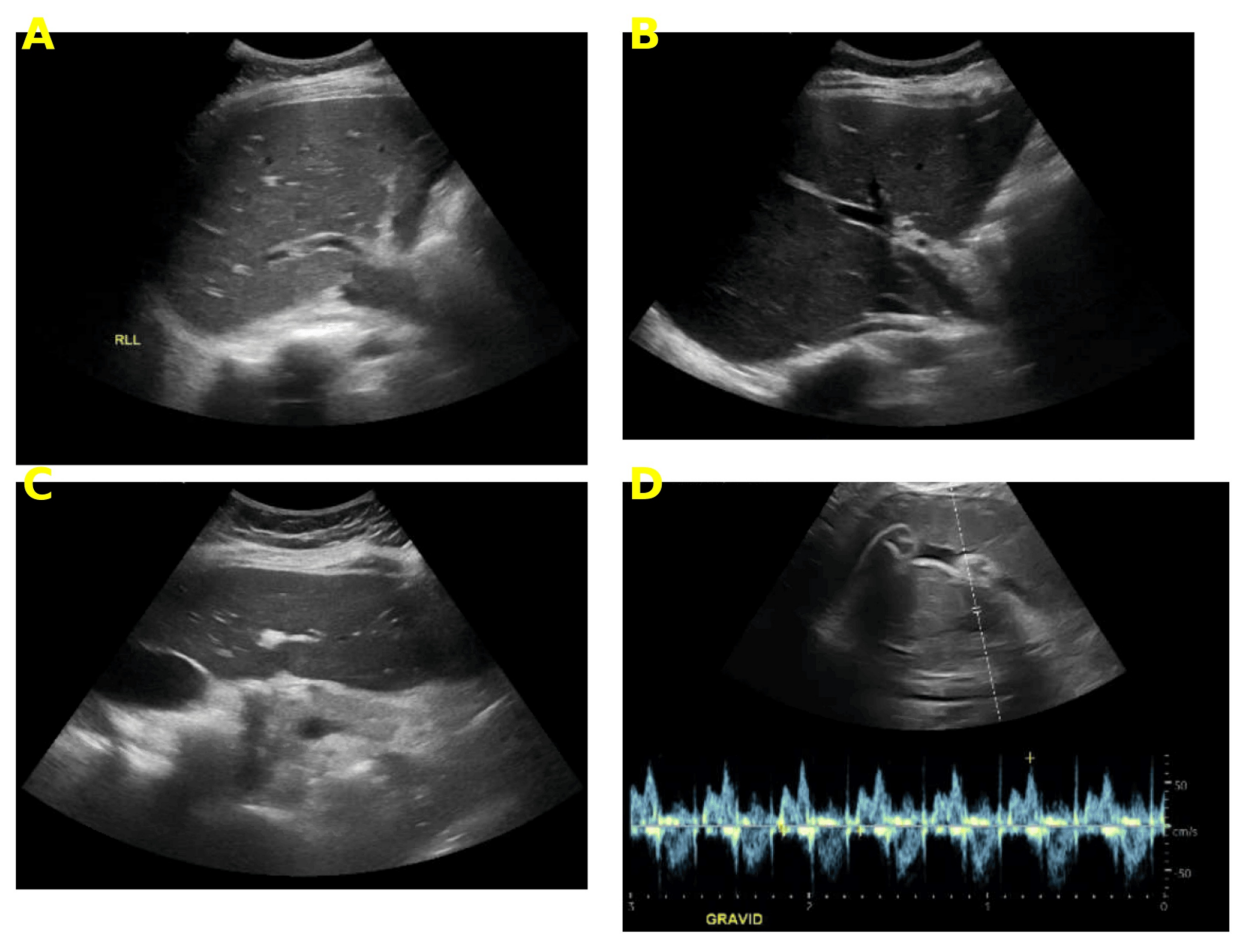
Abdominal ultrasonographic (A) Liver with homogeneous echotexture and smooth contour. No intrahepatic focal lesions or free peritoneal fluid are identified. (B) Longitudinal sonographic view of the gallbladder and biliary tree. The gallbladder appears normal with no wall thickening, calculi, or pericholecystic fluid. The common bile duct is not dilated. (C) Epigastric ultrasound with partial visualization of the pancreas, which appears hyperechoic and without focal lesions. (D) Transabdominal obstetric ultrasound confirms a viable intrauterine pregnancy with a Doppler waveform demonstrating fetal cardiac activity.

On admission, her vital signs were a blood pressure of 142/99 mmHg, heart rate of 110 beats per minute, respiratory rate of 22 per minute, temperature of 36.7 °C, and oxygen saturation of 98% on room air. Early blood pressure readings during her ICU stay were labile, reaching as high as 170/110 mmHg during episodes of clinical deterioration, before stabilization with intravenous labetalol and magnesium sulfate. Abdominal examination revealed marked tenderness in the epigastric region without peritoneal signs or abdominal wall ecchymosis. Obstetric evaluation confirmed a viable intrauterine pregnancy (Figure 1), and there was no peripheral edema.

Initial laboratory evaluation on admission (Table 1) showed profound anemia (hemoglobin 6.3 g/dL), severe thrombocytopenia (platelet count 26 ×10³/μL), and evidence of hemolysis with elevated LDH (1464 U/L) and indirect hyperbilirubinemia (total bilirubin 34 μmol/L; direct 19 μmol/L; indirect 15 μmol/L). Additional findings included acute kidney injury (creatinine 143 μmol/L), high inflammatory markers (CRP 469 mg/L), metabolic acidosis (bicarbonate 13 mmol/L), and markedly increased pancreatic enzymes (amylase 139 U/L, lipase 400 U/L). Coagulation studies showed a prolonged prothrombin time (PT)/international normalized ratio (INR) and elevated D-dimer. Peripheral blood film revealed microangiopathic hemolysis with the presence of schistocytes. ADAMTS13 activity was within the normal range, effectively excluding thrombotic thrombocytopenic purpura.

**Table 1:** Evolution of key laboratory parameters post-delivery AST: aspartate aminotransferase; LDH: lactate dehydrogenase

Day	Hemoglobin (g/dL) (12–16)	Platelets (×10³/μL) (150–400)	Creatinine (μmol/L) (45–90)	AST (U/L) (5–40)	LDH (U/L) (140–280)	Amylase (U/L) (25–125)	Lipase (U/L) (13–60)	CRP (mg/L) (<5)	Bicarbonate (mmol/L) (22–28)
Day 7	12.9	490	39	15	300	32	98	3	25
Day 6	10.5	320	50	18	400	50	120	20	24
Day 5	9	150	60	22	600	70	150	50	23
Day 4	8.8	100	85	32	900	80	200	100	21
Day 3	7.6	60	100	42	1170	100	250	200	18
Day 2	7	50	110	58	1250	120	300	300	15
Day 1	6.3	26	143	71	1464	139	400	469	13

Bedside abdominal ultrasonography demonstrated a normal liver and biliary system, with no evidence of gallstones, biliary dilatation, or pericholecystic fluid (Figure 1). The pancreas was partially visualized and appeared hyperechoic, but further assessment was limited by the gravid uterus. Fetal ultrasound confirmed viability and appropriate cardiac activity. As the diagnosis of acute pancreatitis was established based on laboratory criteria and clinical presentation, and in view of the patient’s pregnancy and hemodynamic instability, advanced imaging, such as MRI or MRCP, was considered but ultimately deferred, as it would not have changed acute management. Biliary and metabolic causes for pancreatitis were excluded by imaging and laboratory assessment.

The patient was admitted to the intensive care unit for thorough monitoring and supportive management, including correction of the electrolytes (potassium, magnesium, and bicarbonate), and supportive transfusion of packed red blood cells and platelets for severe anemia and thrombocytopenia. She was started on broad-spectrum antibiotics (piperacillin-tazobactam), which were escalated to tigecycline due to worsening inflammatory markers. Antihypertensive therapy was optimized with intravenous labetalol, and magnesium sulfate was administered for seizure prophylaxis. Betamethasone was given to facilitate fetal lung maturation.

Despite these interventions, her condition deteriorated with worsening hypertension, evolving multiorgan dysfunction, and signs of fetal distress. After a multidisciplinary team discussion (ICU, Obstetrics, Hematology, Anesthesia, Neonatology), informed consent was obtained, and the patient underwent emergency cesarean section under general anesthesia. A live male infant weighing 340 grams was delivered, with Apgar scores of 2, 6, and 6 at 1, 5, and 10 minutes, respectively. The uterus was preserved, and there were no excessive intraoperative complications. Given her rapid clinical improvement following delivery, genetic testing for atypical hemolytic uremic syndrome (aHUS) was not pursued, and there was no relevant family history or clinical suspicion to drive further complement studies.

**Figure 2:**
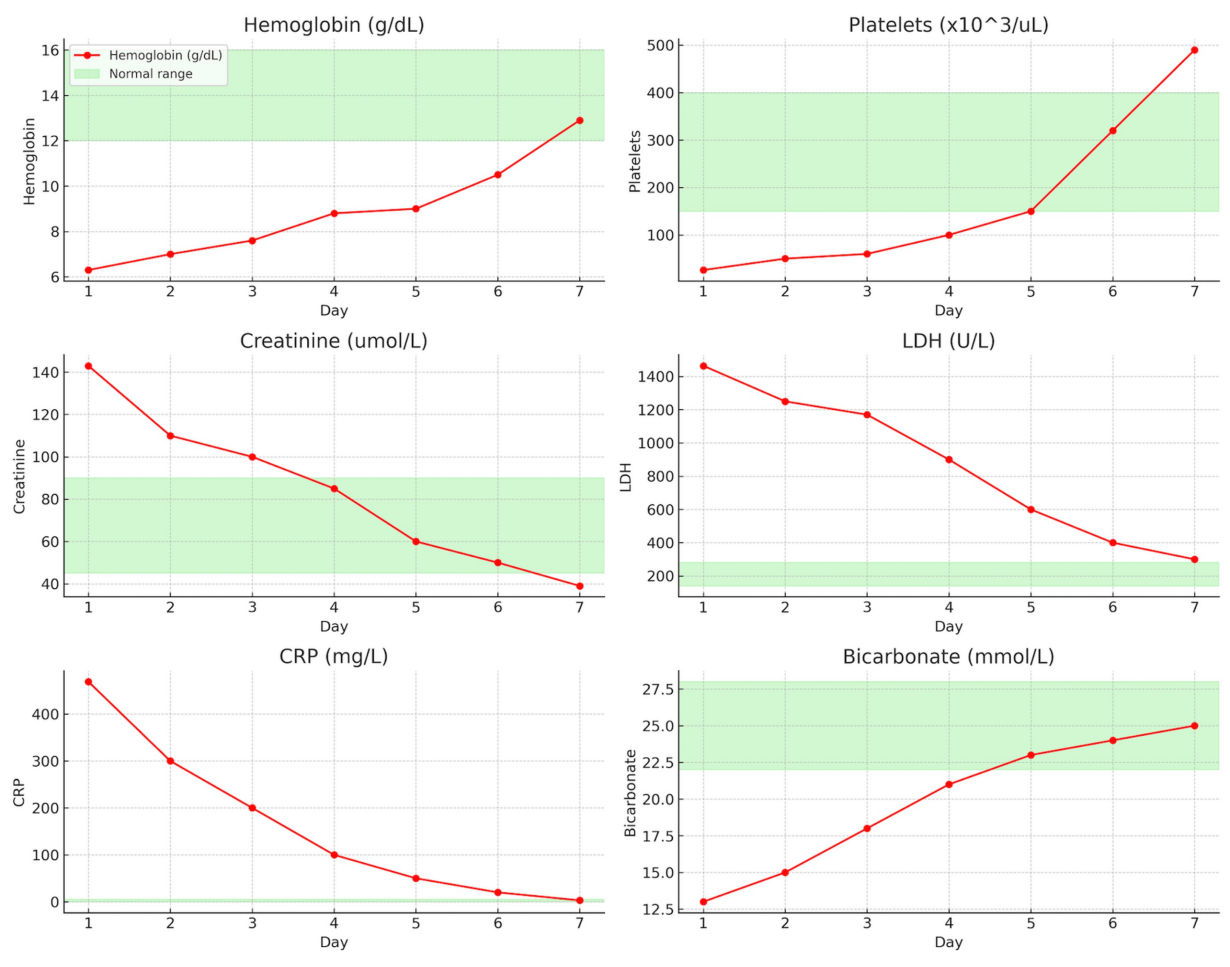
Time course plot showing the progression of laboratory parameters from Day 1 to Day 7 post delivery

Postoperatively, the neonate was admitted to the neonatal intensive care unit for advanced support due to extreme prematurity and low birth weight, requiring mechanical ventilation for respiratory distress. Maternal recovery was complicated by type I respiratory failure, hypertensive pulmonary edema, and diffuse alveolar hemorrhage, which were managed with ventilatory support, careful hemodynamic control, and corticosteroids. Evaluation for posterior reversible encephalopathy syndrome was negative. Over the subsequent days, the patient’s hematological, renal, and pancreatic parameters gradually normalized (Figure 2), allowing transition to a high-dependency unit and eventual discharge in stable condition.

## Discussion

The co-occurrence of HELLP syndrome and acute pancreatitis during pregnancy is exceptionally rare, yet it dramatically increases morbidity for both the mother and fetus. In most reported cases, the clinical presentation is dominated by abdominal pain and multiorgan dysfunction, which can obscure the diagnosis unless there is a high index of suspicion [3,4]. In our patient, classic features of HELLP syndrome, including microangiopathic hemolytic anemia, thrombocytopenia, and hepatic dysfunction, were accompanied by signs of acute pancreatitis, confirmed by elevated amylase and lipase. Notably, common causes of pancreatitis in pregnancy, such as biliary disease and hypertriglyceridemia, were excluded by imaging and laboratory assessment. This supports the hypothesis that severe preeclampsia and HELLP syndrome are characterized by widespread endothelial dysfunction, vasoconstriction, and increased vascular permeability. Although hepatic and renal complications are well-recognized, the pancreas is rarely affected, and end-organ ischemia can extend to pancreatic tissue, precipitating pancreatitis [7,8]. Differential diagnoses, including thrombotic thrombocytopenic purpura (TTP), atypical hemolytic uremic syndrome (aHUS), and malignant hypertension-related thrombotic microangiopathy (TMA), were considered. ADAMTS13 activity was within normal limits, effectively ruling out TTP. Although a genetic screen for aHUS was not performed due to rapid clinical improvement, such testing may be warranted in refractory cases, given the potential overlap in presentation and pathogenesis [9]. Imaging was limited to ultrasonography due to gestational age. Magnetic resonance imaging (MRI) or magnetic resonance cholangiopancreatography (MRCP) was considered but deferred given the diagnostic clarity provided by laboratory data and the clinical urgency. In the absence of biliary pathology on ultrasound and normal triglyceride levels, biliary and metabolic causes were ruled out. Management of these complex cases necessitates early and coordinated multidisciplinary intervention. Supportive critical care, rapid correction of coagulopathy and electrolyte imbalances, antihypertensive therapy, and seizure prophylaxis are foundational. The decision to proceed with urgent delivery was made in the context of the deteriorating maternal status and escalating fetal risk strategy, supported by published literature for severe maternal compromise [2,10,11]. The postpartum period was complicated by type I respiratory failure and diffuse alveolar hemorrhage, attributed to a combination of severe hypertension, HELLP-related endothelial injury, and coagulopathy. The patient’s subsequent stabilization and recovery underscore the benefit of early recognition, a multidisciplinary approach, and aggressive supportive management.

This case contributes to the growing body of evidence suggesting that clinicians should maintain a high degree of suspicion for acute pancreatitis in pregnant women with HELLP syndrome who develop atypical abdominal pain and unexplained multiorgan dysfunction. Early recognition and a tailored multidisciplinary approach remain the cornerstones of optimizing outcomes.

## Conclusions

The concurrence of HELLP syndrome and acute pancreatitis in pregnancy, particularly with added comorbidities, is rare but carries a significant maternal-fetal risk. Early recognition, intensive multidisciplinary management, and timely delivery are key to optimizing the outcomes. This case highlights the necessity for clinical suspicion and collaborative care in pregnant women presenting with abdominal pain and multi-organ dysfunction, especially in the presence of preeclampsia.
